# Supplementary material for: An open-source framework for large-scale, flexible evaluation of biomedical text mining systems
Source: J Biomed Discov Collab. 2008 Jan 29;3:1. doi: 10.1186/1747-5333-3-1 (PMC2276192; doi:10.1186/1747-5333-3-1)
Supplement: Additional file 1 — Evaluation platform source code. The additional file contains the software evaluation framework discussed in this paper. The most current release can be downloaded from SourceForge.net [1]. See the accompanying README file for instructions on installation and use. Also included in the distribution is a collection of UIMA wrappers for some commonly used BioNLP tools and annotated corpora. [file 1747-5333-3-1-S1.zip › bionlp_uima_repository-1.0.1/docs/api/src-html/edu/uchsc/ccp/uima/ae/util/filter/ClassMentionRemovalFilter_AE.html]

```
001    package edu.uchsc.ccp.uima.ae.util.filter;
002    
003    import java.util.ArrayList;
004    import java.util.Iterator;
005    import java.util.List;
006    
007    import org.apache.uima.analysis_engine.ResultSpecification;
008    import org.apache.uima.analysis_engine.annotator.AnnotatorConfigurationException;
009    import org.apache.uima.analysis_engine.annotator.AnnotatorContext;
010    import org.apache.uima.analysis_engine.annotator.AnnotatorContextException;
011    import org.apache.uima.analysis_engine.annotator.AnnotatorInitializationException;
012    import org.apache.uima.analysis_engine.annotator.AnnotatorProcessException;
013    import org.apache.uima.analysis_engine.annotator.JTextAnnotator_ImplBase;
014    import org.apache.uima.jcas.JCas;
015    
016    import edu.uchsc.ccp.uima.annotation.CCPTextAnnotation;
017    import edu.uchsc.ccp.uima.mention.CCPClassMention;
018    
019    /**
020     * removes all annotations that are not a specific class mention from the CAS indexes
021     * @author williamb
022     *
023     */
024    public class ClassMentionRemovalFilter_AE extends JTextAnnotator_ImplBase {
025    
026        public static final String PARAM_CLASS_MENTION_TYPES_TO_REMOVE_LIST = "ClassMentionTypesToRemoveList";
027            
028        private List<String> classMentionTypesToRemove;
029        
030        @Override
031        public void initialize(AnnotatorContext ac) throws AnnotatorInitializationException, AnnotatorConfigurationException {
032            /* read in input parameters, and initialize a list of class mention types to remove */
033            try {
034                String[] classMentionTypes = (String[]) ac.getConfigParameterValue(PARAM_CLASS_MENTION_TYPES_TO_REMOVE_LIST);
035                classMentionTypesToRemove = new ArrayList<String>();
036                for (String type : classMentionTypes) {
037                    classMentionTypesToRemove.add(type.toLowerCase());
038                }
039            } catch (AnnotatorContextException e) {
040                e.printStackTrace();
041            }
042            
043            System.err.println("Initialized ClassMentionRemovalFilter; types to remove: " + classMentionTypesToRemove.toString());
044            
045            super.initialize(ac);
046        }
047        
048        
049        /**
050         * cycle through all annotations and set the annotation set
051         */
052        public void process(JCas jcas, ResultSpecification arg1) throws AnnotatorProcessException {
053            
054            List<CCPTextAnnotation> annotationsToRemove = new ArrayList<CCPTextAnnotation>();
055            
056            Iterator annotIter = jcas.getJFSIndexRepository().getAnnotationIndex(CCPTextAnnotation.type).iterator();
057    
058            while (annotIter.hasNext()) {
059                Object possibleAnnot = annotIter.next();
060                if (possibleAnnot instanceof CCPTextAnnotation) {
061                    CCPTextAnnotation ccpTA = (CCPTextAnnotation) possibleAnnot;
062                    CCPClassMention ccpCM = ccpTA.getClassMention();
063                    String classMentionType = ccpCM.getMentionName().toLowerCase();
064                    if (classMentionTypesToRemove.contains(classMentionType)) {
065                        annotationsToRemove.add(ccpTA);
066                    }
067                } else {
068                    warn("CCPTextAnnotation expected but instead got " + possibleAnnot.getClass().getName());
069                }
070            }
071            
072            /* now remove annotations that had class mention types in the classMentionTypesToRemove list */
073            int count = 0;
074            for (CCPTextAnnotation ccpTA : annotationsToRemove) {
075                ccpTA.removeFromIndexes();
076    //            ccpTA = null;
077                count++;
078            }
079            System.err.println("ClassMentionRemovalFilter Removed " + count + " annotations.");
080    
081        }
082    
083        
084        private void warn (String message) {
085            System.err.println("WARN -- ClassMentionRemovalFilter: " + message);
086        }
087    }
```
